# Recent developments in application of single-cell RNA sequencing in the tumour immune microenvironment and cancer therapy

**DOI:** 10.1186/s40779-022-00414-y

**Published:** 2022-09-26

**Authors:** Pei-Heng Li, Xiang-Yu Kong, Ya-Zhou He, Yi Liu, Xi Peng, Zhi-Hui Li, Heng Xu, Han Luo, Jihwan Park

**Affiliations:** 1 Department of Thyroid and Parathyroid Surgery, Laboratory of Thyroid and Parathyroid Disease, Frontiers Science Centre for Disease-Related Molecular Network, Department of General Surgery, West China Hospital, Sichuan University, Chengdu, 610044 China; 2 Department of Oncology, West China School of Public Health and West China Fourth Hospital, Sichuan University, Chengdu, 610044 China; 3 Department of Rheumatology and Immunology, Rare Diseases Centre, West China Hospital, Sichuan University, Chengdu, 610044 China; 4 College of Computer Science, Sichuan University, Chengdu, 610065 China; 5 State Key Laboratory of Biotherapy and Cancer Centre, West China Hospital, Sichuan University and Collaborative Innovation Centre, Chengdu, 610044 China; 6 School of Life Sciences, Gwangju Institute of Science and Technology (GIST), Gwangju, 61005 Republic of Korea

**Keywords:** Single-cell RNA sequencing (scRNA-seq), Tumour immune microenvironment (TIME), Trajectory, Cellular interactions, Therapeutic targets

## Abstract

The advent of single-cell RNA sequencing (scRNA-seq) has provided insight into the tumour immune microenvironment (TIME). This review focuses on the application of scRNA-seq in investigation of the TIME. Over time, scRNA-seq methods have evolved, and components of the TIME have been deciphered with high resolution. In this review, we first introduced the principle of scRNA-seq and compared different sequencing approaches. Novel cell types in the TIME, a continuous transitional state, and mutual intercommunication among TIME components present potential targets for prognosis prediction and treatment in cancer. Thus, we concluded novel cell clusters of cancer-associated fibroblasts (CAFs), T cells, tumour-associated macrophages (TAMs) and dendritic cells (DCs) discovered after the application of scRNA-seq in TIME. We also proposed the development of TAMs and exhausted T cells, as well as the possible targets to interrupt the process. In addition, the therapeutic interventions based on cellular interactions in TIME were also summarized. For decades, quantification of the TIME components has been adopted in clinical practice to predict patient survival and response to therapy and is expected to play an important role in the precise treatment of cancer. Summarizing the current findings, we believe that advances in technology and wide application of single-cell analysis can lead to the discovery of novel perspectives on cancer therapy, which can subsequently be implemented in the clinic. Finally, we propose some future directions in the field of TIME studies that can be aided by scRNA-seq technology.

## Background

Since the beginning of the twenty-first century, tumours have been a great threat to human health. Tumour heterogeneity has a significant impact on cancer prognosis and response to therapies [1]. Traditional genomic and transcriptomic analyses have been widely used to study different cancer types, stratifying tumours into distinct groups. Some previous findings have been translated into clinical practice owing to their potential roles in predicting prognosis and response to different therapies, as well as providing targets for cancer therapy [2–6]. However, previous studies have mainly focused on the genomic and transcriptomic features of malignant cells. In addition to malignant cells, the tumour immune microenvironment (TIME) is an essential component of various tumours. Single-cell profiling of different cancer types indicates that the cellular details of the TIME are primarily shared across multiple cancer types [7]. Currently, the TIME has drawn increasing attention since the discovery of checkpoint inhibitors, and immunotherapy has revolutionized cancer treatment [8, 9]. The TIME is composed of noncellular components (vessels, extracellular matrix, signaling molecules, etc.) and cellular components (T cells, myeloid cells, fibroblasts, etc.) [10–12]. Although traditional genomic and transcriptomic analyses, such as CIBERSORTx and DWLS, also emphasize immune-related pathways and computational approaches and have been applied to predict immune cell components, technical limitations have confounded the precise characterization of the TIME [13, 14].

Traditional bulk genomic and transcriptomic analyses average signals from a group of different cells, which obscures the identification of specific cell types and states. In situ hybridization and immunohistochemistry have been used to explore the genomic, transcriptomic, and proteomic characteristics of individual cells, but their outputs are relatively low [15, 16]. Flow cytometry and cytometry by time-of-flight (CyTOF) are capable of analysing thousands or millions of single-cell proteomic profiles [17]; however, these methods require prior selection of antibodies of interest. With breakthroughs in cell isolation and sequencing technologies, single-cell RNA sequencing (scRNA-seq) has enabled unbiased genome-wide profiling of many cells at the single-cell level in a single run. scRNA-seq has been used to analyse the transcriptomics of individual cells, which helps characterize the cellular heterogeneity in each sample [18–21].

In this review, we mainly focus on the TIME, which plays an essential role in tumorigenesis, as well as in cancer progression, invasion, and metastasis [22]. The TIME has shown potential in diagnosing, treating, and predicting the prognosis of different types of cancer [23]. Compared with conventional methods, scRNA-seq can be utilized to identify novel cellular types and corresponding cellular states, deepening our understanding of TIME [24]. Furthermore, combining scRNA-seq with other computational methods can reveal dynamic changes in the TIME. Hence, here, we review new findings concerning the TIME discovered through the application of scRNA-seq.

## Application of scRNA-seq in TIME and cancer therapy

### Technological advances in scRNA-seq

**Fig. 1:**
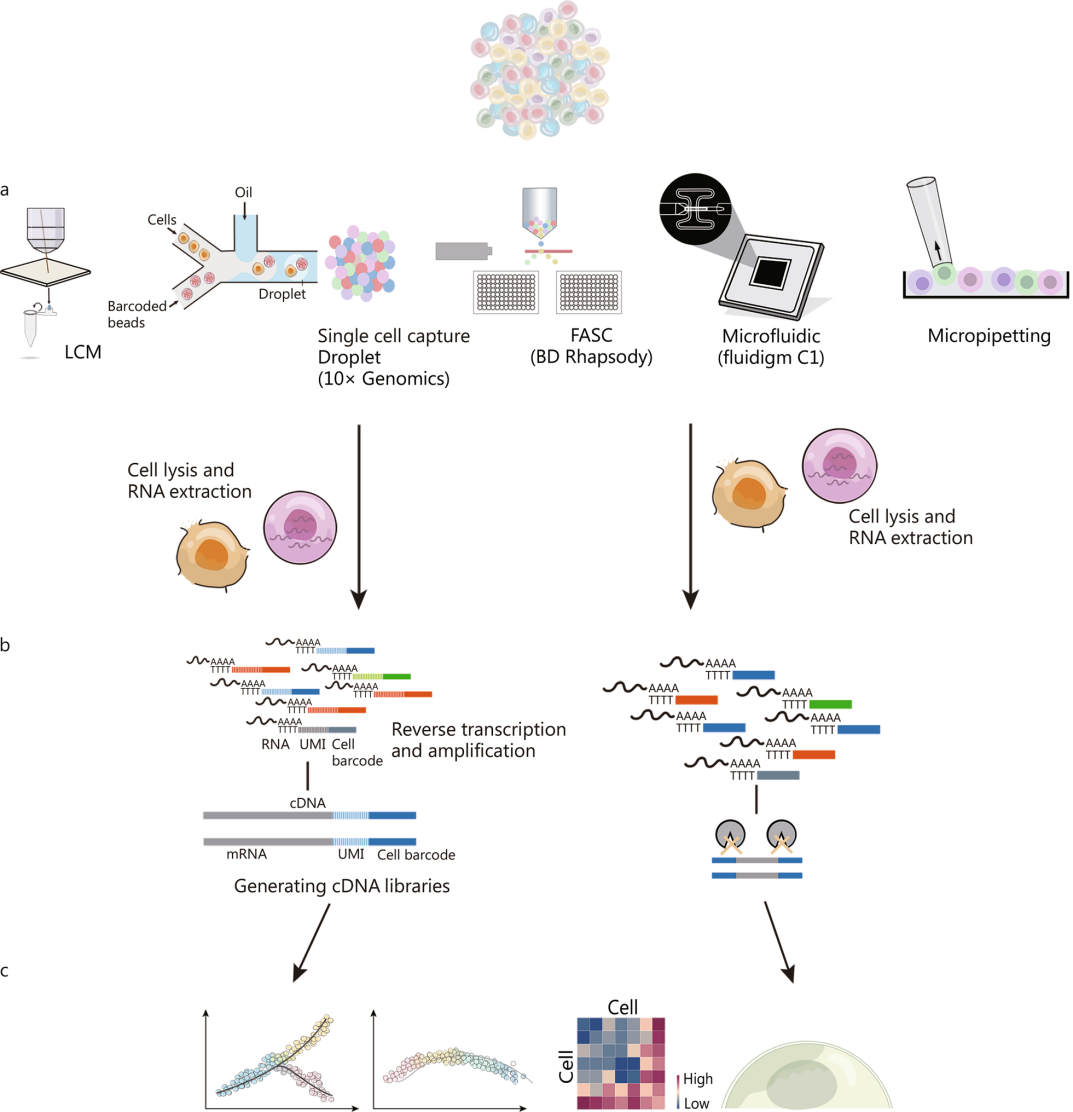
The main procedure for single-cell RNA sequencing. The main procedure for single-cell RNA sequencing includes separation and extraction of single cells (**a**), reverse transcription amplification, generation of cDNA libraries, sequencing (**b**) and Data analysis (**c**). FASC fluorescence-activated cell sorting, UMI unique molecular identifier, cDNA complementary DNA

The main scRNA-seq procedure includes separation and extraction of single cells, cDNA synthesis, nucleic acid amplification, sequencing, and data analysis [25]. We depict the major procedures of scRNA-seq in Fig. 1. One challenge of scRNA-seq is the relatively small amount of RNA in individual cells compared with traditional bulk sequencing. Thus, more efficient amplification methods are needed. Researchers have successfully established stable single-cell library construction processes to generate sufficient cDNA for sequencing, including polymerase chain reaction (PCR) amplification, such as SMART-Seq2, and in vitro transcription (IVT) amplification, such as CEL-Seq2 [26].

Single-cell separation and capture are essential procedures for scRNA-seq in different approaches. Figure 1a concludes the current common approaches for single-cell separation and capture. These procedures fall into four major categories: laser capture microdissection (LCM), micromanipulation, fluorescence-activated cell sorting (FACS), and microfluidics [27, 28]. Fluidigm C1, launched in 2012, was the first commercial platform using microfluidic chips. Taking advantage of Fluidigm C1 lowers the threshold for single-cell sequencing [29]. The Fluidigm C1 microfluidics system allows researchers to obtain the complete transcriptome data of 96 cells with diameters of 5–25 μm in a single run. This approach also provides high-quality gene expression readouts. However, low cell throughput and high cost limit its application. Thus, this approach is mainly used for small-sample studies [30, 31].

The development of microfluidics and reverse emulsion devices allows the isolation of single cells into droplets, which is currently the most widely used method. The approach is exemplified by two academically developed technologies, Drop-seq and inDrop [32, 33]. Compared with Drop-seq, inDrop is easier to set up and produces higher throughput data as it detects more genes. However, inDrop cell barcoding has a much higher error rate than Drop-seq and higher reagent costs [34]. In 2017, the commercial sequencing platform 10 × Genomics was successfully developed based on the above techniques, enabling a significant increase in cell throughput and a considerable reduction in single-cell sequencing costs [35].

In addition to microfluidic devices trapping cells inside hydrogel droplets, FACS combined with microwell plates is a commonly applied scRNA-seq technique [36]. Various amplification methods can be used within microwell plates, including SMART-seq2, CEL-Seq, MARS-seq, and STRT-seq [37–40]. These manual approaches are not restricted by the cell size constraints of the Fluidigm system, and the equipment and setup costs are meagre. More importantly, SMART-seq2 and STRT-seq allow for full coverage of cDNA sequencing, facilitating the analysis of alternative splicing patterns [41]. In contrast, Drop-seq and inDrop only provide sequence information at the 3′ or 5′ ends of the cDNA. Figure 1b displays the reverse transcription amplification and generation of cDNA libraries of different approaches. CytoSeq also takes advantage of microwells for cell separation and capture. A bead similar to that in the Drop-seq approach is suspended in each well using CytoSeq. Compared with Drop-seq, CytoSeq does not require a complicated microfluidic system to sort cells, and it is easier to achieve sequencing throughput expansion using plates with more microwells [42]. Derived from CytoSeq and launched in 2018, the BD Rhapsody platform uses a large number of microwells, far exceeding the number of cells for cell capture, to ensure that each cell occupies a single well [43].

The advent of SPLiT-seq reduced the cost of library construction for sequencing to $0.01 for each cell. SPLiT-seq reduces equipment requirements and the costs for generating cDNA libraries by labelling the cellular origin of RNA through combinatorial barcoding. In addition, the quality of scRNA-seq data obtained was similar to that obtained with Drop-seq and InDrop [44]. The common down-stream data analysis of scRNA-seq is shown in Fig. 1c. Table 1 compares the current major scRNA-seq technologies. The principles, number of cells in each single-run and sensitivity of different approaches are concluded. In addition, we also reviewed the strength and weakness of each approaches, as well as their potential applications.

**Table 1:** Comparison of the characteristics and applications of different scRNA-seq technologies

Characteristics and applications	Drop-Seq, In-Drop	STRT-seq, SMART-seq2	CEL-seq, MARS-seq	CytoSeq	SPLiT-seq	Microchip
Approaches	Microdroplet approaches	Microwell plate-based approaches	Microwell plate-based approaches	Microwell plate-based approaches	Other approaches	Microfluidics
Single cell separation and capture	Microdroplets	FACS	FACS	FACS	None	Micofluidics
Number of cells in a single run	5000–10,000	50–500	500–2000	100–10,000	> 100,000	48–96
Sensitivities of gene detection	Cell line: 5000 genes per cell Tissue: 1000–3000 genes per cell	Cell line: 7000–10,000 genes per cell Tissue: 2000–6000 genes per cell	Cell line: 7000–10,000 genes per cell Tissue: 2000–6000 genes per cell	Cell line: 7000–10,000 genes per cell Tissue: 2000–6000 genes per cell	Cell line: 7000–10,000 genes per cell Tissue: 2000–6000 genes per cell	Cell line: 6000–9000 genes per cell@@@Tissue: 1000–5000 genes per cell
Strength	High throughput; Low cost; Easy to operate; High degree of automation	Flexible optimization; Low requirements for cell sample size; Many quality control points; Full cDNA sequencing; High sensitivity of gene detection		Low cost; High throughput; Conducive to targeted capture	Low cost; High throughput; Larger number of samples processed at one time; High capture rate; Low requirements for sample preparation	Capture maximum 96 cells in a single run; Low technical requirements; Short experiment period; Full cDNA sequencing; High sensitivity of gene detection
Weakness	High requirements for cell number and activity; 3’ sequencing instead of full cDNA sequencing; Lack of quality control points	Manual operation; High technical requirements; Low throughput and high cost; Time consuming		High requirement for cell number; 3’ sequencing instead of full cDNA sequencing; Complicated procedure	3’ sequencing instead of full cDNA sequencing	Lack of quality control points; Low throughput; High costs
Application	Clustering and lineage determination in large-scale cell samples; Cells with diameter < 40 µm	Experienced researchers; In-depth analysis of small sample size		Clustering and lineage determination in large-scale cell samples; Cells with diameter < 20 µm	Clustering and lineage of large-scale cell sample; In-depth analysis of small sample size	In-depth analysis of small sample size Cells with diameter of 5–25 μm
Commercial platform	10 × Genomics	–		BD rhapsody	–	Fluidigm C1

“–” not applicable. *FACS* fluorescence-activated cell sorting

Future development of scRNA-seq techniques may reduce the costs and increase the cell throughput, making scRNA-seq a standard tool for studying individual cell transcriptomes. Genomes of individual cells can be inferred from computational approaches [45] or from newly developed single-cell sequencing approaches [46]. In addition, single-cell protein expression and epigenomics can also be analysed to better understand cellular diversity and gene regulatory mechanisms. Recent reviews have concluded that the advances in these technologies [47, 48] still need improvement before wide application. High-throughput single-cell multiomics data might play an essential role in uncovering the features of individual cells at unprecedented resolution in the future.

As scRNA-seq technology becomes widespread, specialized computational methods and tutorials for scRNA-seq data analysis have been put forward [49]. (1) The batch effect is a common issue in data integration. Several batch-effect correction methods have been utilized, such as Harmony [50], fastMNN [51], and Scanorama [52], and a recent study compared the properties of these methods [53]. (2) scRNA-seq data are high dimensional because each cell contains the expression of more than 10,000 genes (variables). Dimensionality reduction is required to improve downstream analysis. Different approaches have been proposed, such as principal component analysis (PCA) [54], nonnegative matrix factorization (NMF) [55], and deep neural networks [56]. The detailed features of these methods have been described in other reviews [57, 58]. Zero inflation is another challenge of scRNA-seq data analysis. There are a large number of zero values in the scRNA-seq expression matrix. This is due to stochastic gene expression, the different states of various cells and technical noise, such as RNA capture efficiency and random dropouts during library preparation [59]. Many methods, including MAGIC [60], CIDR [61] and scImpute [62], have been used to impute the zeros in the expression matrix. However, these methods can introduce false-positive results while reducing technical noise. To conclude, due to the complexity of scRNA-seq data, the computational methods still need to be improved.

### Conventional cellular components in the TIME

The cellular components of the TIME include lymphocytes (T and NK cells), myeloid cells (macrophages and dendritic cells), fibroblasts, and other immune cells. Fibroblasts are traditionally categorized as stromal cells due to their essential roles in constructing the extracellular matrix. Here, we include cancer-associated fibroblasts in the TIME, as they secrete abundant proinflammatory and anti-inflammatory factors to reshape the TIME.

Cytotoxic CD8^+^ T cells, which recognize specific antigens on tumour cells and subsequently eliminate them, are the most common and effective immune cells in the TIME [63]. The cytotoxic function of CD8^+^ T cells relies on CD4^+^ T helper 1 (Th1) cells [64]. Other CD4^+^ T cells, including T helper 2 (Th2) cells and T helper 17 (Th17) cells, also facilitate immune responses in the tumour microenvironment [65, 66]. In contrast, regulatory T cells (Tregs) inhibit the TIME and exacerbate tumour progression [67, 68]. Natural killer T (NKT) and natural killer (NK) cells are also involved. Their receptors recognize tumour cells, which leads to the activation of other immune cells [69, 70].

As an important constituent of innate immunity, myeloid cells, including tumour-associated macrophages (TAMs) and dendritic cells (DCs), play essential roles in the TIME. Macrophages are conventionally classified into proinflammatory M1 and anti-inflammatory M2 phenotypes. TAMs populations are predominantly composed of M2 macrophages, facilitating tumour growth, tumour survival, and angiogenesis by producing growth factors and cytokines [71]. However, DCs are essential for antigen presentation to T cells, connecting innate and adaptive immunity [72–74].

Cancer-associated fibroblasts (CAFs) sustain proliferation and secrete regulatory factors in the TIME and can be divided into inflammatory CAFs (iCAFs) and myofibroblastic CAFs (myCAFs). iCAFs have higher secretion of cytokines and chemokines, while myCAFs highly express contractile proteins [75]. CAFs have conflicting effects on the TIME. Some studies have demonstrated that CAFs recruit M2 macrophages and Tregs, inhibiting immune responses in the tumour microenvironment [76, 77]. CAFs have also been found to support antitumor immunity in some cases [78].

In addition to secreting antibodies, B cells also participate in cellular immunity through the production of cytokines that interact with T cells [79]. Studies have shown that B cells inhibit cytotoxic T cells [80] and induce CD4^+^ T cell differentiation into Tregs [81, 82]. Furthermore, B cells are essential components of recently introduced tertiary lymphoid structures (TLSs). B-cell-rich TLSs are associated with survival and immunotherapy responses in various tumours [83, 84].

Previous studies have emphasized the essential role of cellular components in the TIME. However, the identification of immune cells is based on limited cell markers with the aid of immunohistochemistry. Transcriptomic atlases of individual immune cells are required to explore distinct immune cells and their corresponding functions. In addition, cell evolution is a dynamic process in which cellular alterations gradually accumulate. To understand this evolutionary process and its determinants, it is necessary to observe the transcriptomic fluctuation of every single cell simultaneously, which requires the application of scRNA-seq.

### New discoveries concerning the TIME explored by scRNA-seq

Clustering and annotation are essential in interpreting scRNA-seq data and exploring the TIME. The data are partitioned based on cell similarities [85–87], and the challenge is to estimate the intrinsic cluster number or density without providing a priori knowledge [88]. A possible solution is to adopt hierarchical clustering methods to reveal the hierarchical structure of cells [89], which is also consistent with cell ontologies. Given a data partition result produced by clustering methods, cell type annotation is needed to provide biological meanings. The primary challenge of annotation is determining how many cell types are present in each cluster and whether currently undiscovered cell types exist [90]. In practice, researchers typically first identify marker genes of each cluster and then annotate them based on expertise and literature. To avoid subjective annotations, certain tools can automatically annotate cells based on evident or probabilistic similarities, leveraging a wide range of marker repositories as references [91–93]. This variant of annotation methods builds on the transfer learning paradigm [94]. Specifically, a cell type classifier is trained based on previously annotated scRNA-seq data, which can directly map gene expression to cell type [95, 96]. A similar approach is to match query cells with annotated ones to establish correspondences [97, 98]. However, these methods might produce suboptimal results when data are heavily confounded with batch effects; thus, preliminary batch integration would be necessary. Here, we mainly focus on studies that annotated clusters by identifying marker genes based on expertise and the literature.

**Fig. 2:**
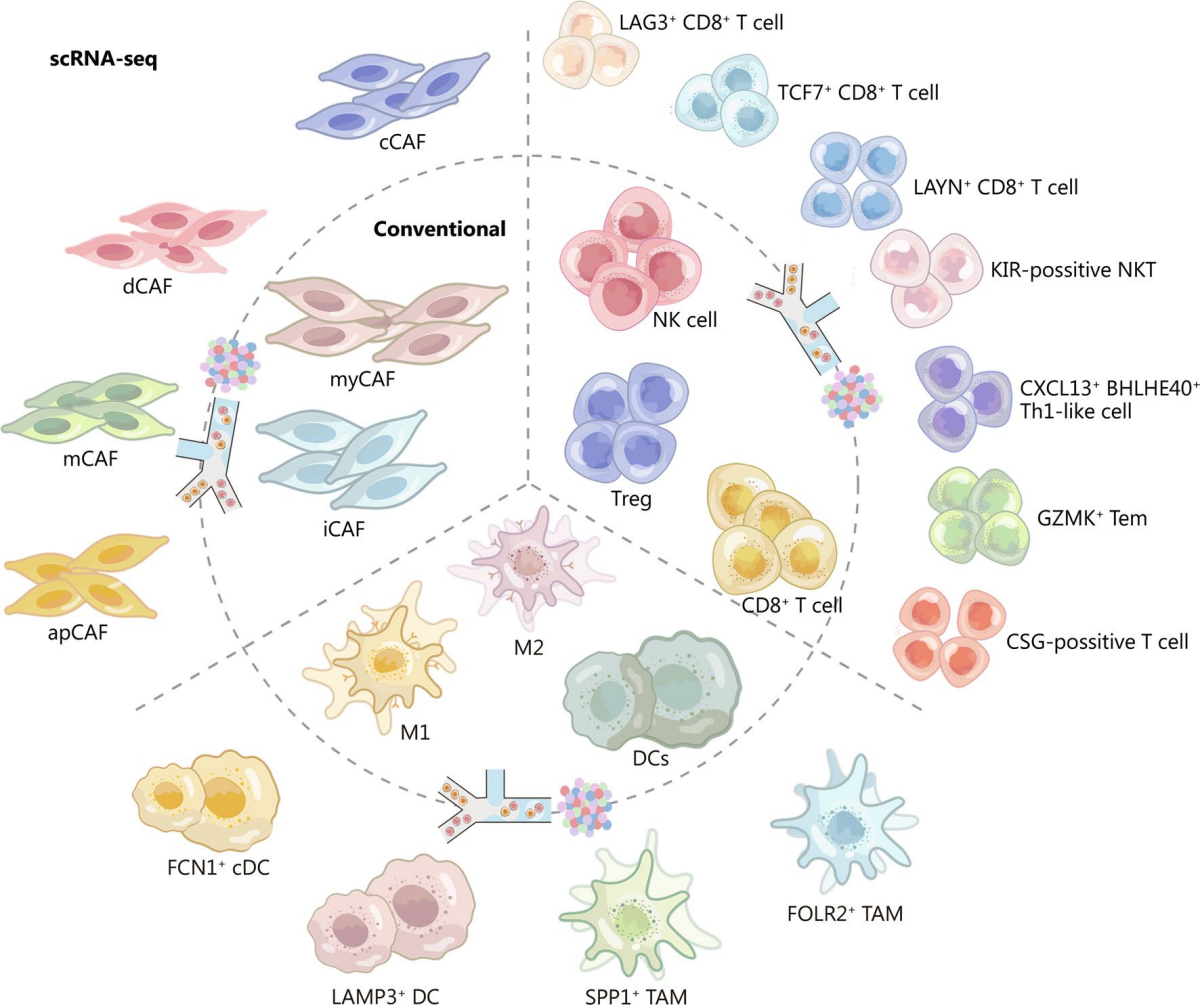
New resolution of immune cell clusters revealed by scRNA-seq. With the help of scRNA-seq, novel cell clusters of CAFs, T cells, TAMs and DCs have been identified. CAFs cancer-associated fibroblasts, cCAFs cycling CAFs, dCAFs developmental CAFs, apCAFs antigen-presenting CAFs, iCAFs inflammatory CAFs, myCAFs myofibroblastic CAFs, mCAFs matrix CAFs, TAMs tumour-associated macrophages, DCs dendritic cells, cDC conventional DC, FCN1 ficolin 1, LAMP3 lysosomal associated membrane protein 3, SPP1 secreted phosphoprotein 1, FOLR2 folate receptor Beta, Tregs regulatory T cells, NK natural killer, CSG cell surface glycoprotein, GZMK granzyme K, CXCL13 C-X-C motif chemokine ligand 13, BHLHE40 basic helix-loop-helix family member E40, Th1 T helper 1, KIR killer cell immunoglobulin like receptor, LAYN layilin, TCF7 transcription factor 7, LAG3 lymphocyte activating 3

scRNA-seq has enabled researchers to classify immune cells into subpopulations with distinct functions at a higher resolution, which depicts the heterogeneity of conventional subtypes of immune cells. Novel clusters of lymphocytes (T and NK cells), myeloid cells (macrophages and dendritic cells), and fibroblasts discovered with the help of scRNA-seq are concluded in Fig. 2. scRNA-seq of human and mouse samples indicated that CAFs could be categorized as antigen presentation CAFs (apCAFs), iCAFs, or myCAFs. apCAFs uniquely express major histocompatibility complex (MHC) class II genes, including CD74, which activates CD4^+^ T cells [99]. A similar subpopulation of apCAFs has also been observed in colorectal cancer [100]. scRNA-seq of fibroblasts in a genetically engineered mouse model of breast cancer further identified vascular CAFs (vCAFs), matrix CAFs (mCAFs), developmental CAFs (dCAFs), and cycling CAFs (cCAFs) [101]. vCAFs, mCAFs, and dCAFs seem to originate in a perivascular location when resident fibroblasts and malignant cells undergo epithelial-mesenchymal transition (EMT) [102]. cCAFs are the proliferating portion of the vCAFs population. vCAFs and mCAFs were also found in other mouse models, were conserved in patient breast tumour samples, and were found to increase the metastasis of breast cancer cells [103]. Improving the resolution of CAFs provides biomarkers for the development of drugs that precisely target CAFs. Another scRNA-seq study of breast cancer divided Tregs into five clusters: Tregs coexpressing Cytotoxic T lymphocyte-associated antigen-4 (CTLA-4), T cell immunoreceptor with Ig and ITIM domains (TIGIT), and GITR and other Tregs mutually or exclusively expressing the same genes, which presented distinct functions [104]. Patients with different prognoses have different proportions of Treg clusters, providing targets for personalized therapy. More detailed pan-cancer research focusing on the T cells and myeloid cells in the TIME revealed the existence of granzyme K (GZMK)^+^ Tem, interferon-stimulated genes (ISG)^+^ T cells, killer cell immunoglobulin like receptor (KIR)^+^ NKT cells, transcription factor 7 (TCF7)^+^ CD8^+^ T cells, ficolin 1 (FCN1)^+^ conventional DC (cDC)2, secreted phosphoprotein 1 (SPP1)^+^ TAMs, and folate receptor beta (FOLR2)^+^ TAMs in the tumour microenvironment [105, 106].

Based on scRNA-seq data, novel subpopulations of immune cells have also been discovered in the TIME. scRNA-seq of uveal melanoma identified previously unrecognized cell types, including CD8^+^ T cells that predominantly express the checkpoint marker LAG3 instead of programmed death-1 (PD-1) or CTLA-4 [107]. Meanwhile, clonal enrichment of infiltrating exhausted CD8^+^ T cells and Tregs with high expression of layilin (LAYN) was found in hepatocellular carcinoma [108]. These studies provide novel targets for cancer immunotherapy, as CD8^+^ T cells are the main constituents involved in elimination of malignant cells. scRNA-seq of colorectal cancer identified C-X-C motif chemokine ligand (CXCL)13^+^ basic helix-loop-helix family member E40 (BHLHE40)^+^ Th1-like cells associated with the interferon-γ (IFN-γ)-regulating transcription factor BHLHE40. These cells were found to have a favourable response to immune checkpoint blockade in microsatellite-instable tumours [109], potentially increasing the efficacy of immunotherapy.

DCs are essential for presenting antigens to activate T cells in the TIME. scRNA-seq of gastric cancer revealed a novel DCs cluster expressing indoleamine 2,3-dioxygenase 1 (IDO1) and the chemokines C–C motif chemokine ligand (CCL)22, CCL17, CCL19, and interleukin (IL)-32, which are involved in the recruitment of T cells [110]. scRNA-seq of pancreatic ductal adenocarcinoma also identified DCs clusters that highly expressed IDO1 in addition to conventional cell markers. IDO1 is essential for catalysing tryptophan depletion and kynurenine production, inhibiting T-cell proliferation and cytotoxicity [99], which reveals the close interactions between DCs and T cells. Moreover, lysosomal associated membrane protein 3 (LAMP3)^+^ DCs were identified by scRNA-seq and appeared to be the mature form of classical DCs. LAMP3^+^ DCs can migrate to lymph nodes and highly express ligands that interact with T cells [111]. The discovery of these novel DCs clusters expressing specific markers provides a new perspective for cancer immunotherapy because of their strong communication with T cells.

Novel signature genes of TAMs, including triggering receptor expressed on myeloid cells 2 (TREM2), CD81, macrophage receptor with collagenous structure (MARCO), and apolipoprotein E (APOE), were discovered in lung adenocarcinoma using scRNA-seq [112]. Furthermore, scRNA-seq of breast cancer indicated that the angiogenesis factors plasminogen activator, urokinase receptor (PLAUR) and IL-8 were expressed in TAMs in addition to M2-type genes, such as CD163, membrane spanning 4-domains A6A (MS4A6A), and transforming growth factor beta-1 (TGF-β1) [113]. These novel gene signature profiles in TAMs are associated with patient survival and provide new potential targets for cancer therapy. scRNA-seq of tumour samples revealed that a subpopulation of TAMs presented with high expression of SPP1, the macrophage scavenger receptor MARCO, and MHC II class genes. MARCO and SPP1 are anti-inflammatory and immune-suppressive signatures in macrophage activation, while MHC II class genes are related to proinflammatory functions [99, 100]. Additional scRNA-seq studies have indicated that TAMs frequently present with both proinflammatory and anti-inflammatory signatures [104]. This phenomenon suggests that macrophage activation in the tumour microenvironment is not consistent with conventional M1/M2 polarization, which is further discussed in the next section.

### Evolution of immune cells suggested by single-cell data

Most immune cells are in the process of cellular development. An abundance of immune cells are in transient states of developmental trajectories rather than the discrete states of well-differentiated cells. With the help of scRNA-seq and in-depth analyses, researchers can explore the characteristics of differentiated cells and the transition of a specific cell type and its possible mechanisms.

The most commonly used computational method is the pseudotime trajectory. The pseudotrajectory describes the developmental processes of cells, characterized by cascading changes in gene expression. A branching point represents a significant divergence in cellular differentiation. Various machine learning computational methods have been utilized to construct trajectories, including Monocle3 [114], DTFLOW [115], DPT [116], SCORPIUS [117], and TSCAN [118], which have been evaluated and compared in a separate review [119]. As TAMs and T cells represent the most abundant immune cell types in the TIME, we mainly focus on these two cell types.

**Fig. 3:**
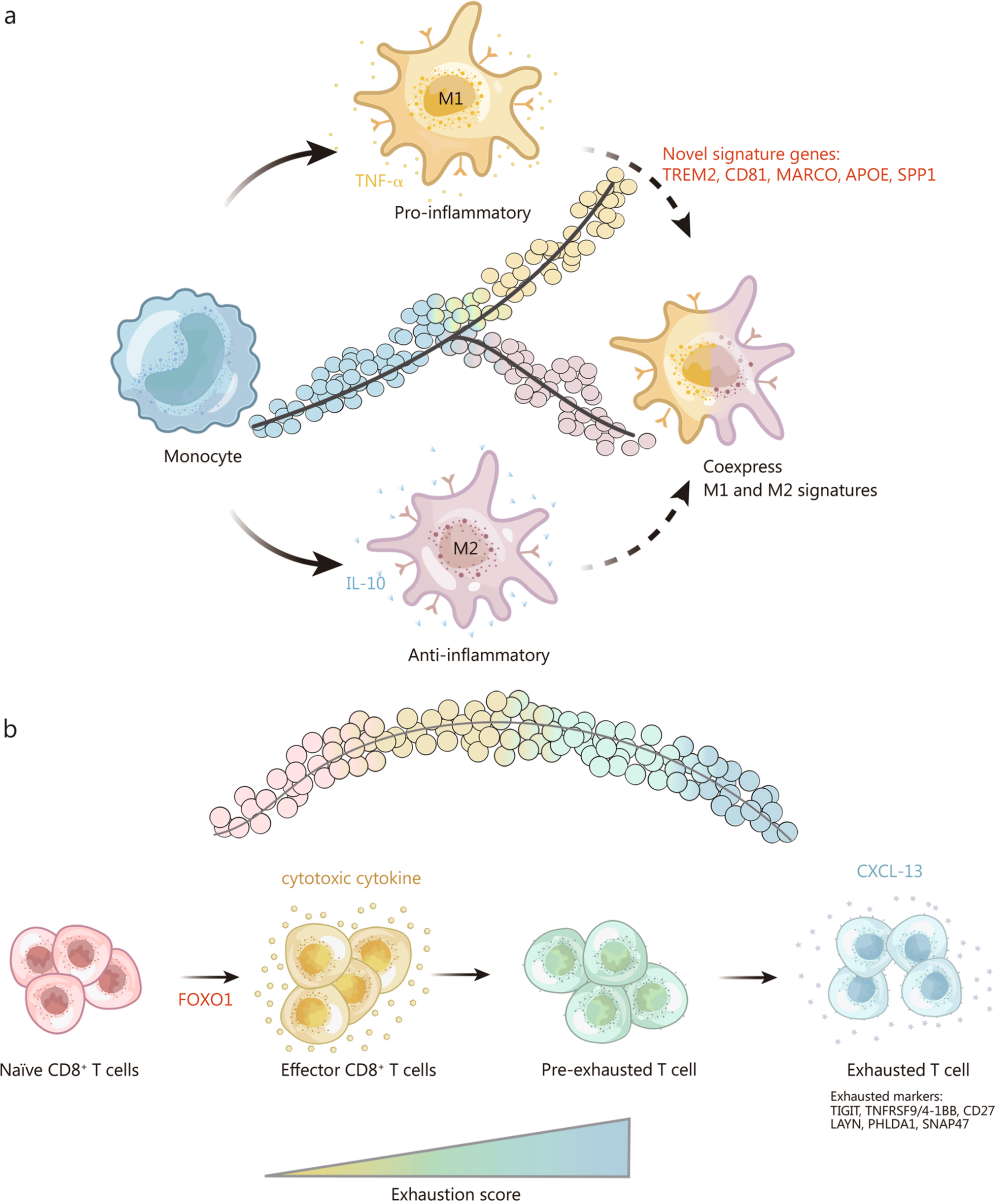
Evolution of tumour-associated macrophages and T cells. **a** Tumour-associated macrophages (TAMs) coexpressing M1 and M2 genes might evolve from M1 or M2 macrophages. **b** Naïve T cells can develop into effector CD8 T cells, which might develop into pre-exhausted T cells and further into exhausted T cells. TNF-α tumor necrosis factor-α, TREM2 Triggering receptor expressed on myeloid cells 2, MARCO macrophage receptor with collagenous structure, APOE apolipoprotein E, SPP1 secreted phosphoprotein 1, IL-10 interlukin-10, FOXO1 forkhead box O1, CXCL13 C-X-C motif chemokine ligand 13, TIGIT T cell immunoreceptor with Ig and ITIM domains, TNFRSF9 TNF receptor superfamily member 9, LAYN layilin, PHLDA1 pleckstrin homology like domain family A member 1, SNAP47 synaptosome associated protein 47

scRNA-seq of various tumours revealed that TAMs frequently coexpress M1 genes, including TNF-α, and M2 genes, such as IL-10 [104], and that the differentiation and states of TAMs are directly correlated with their antitumor effects. Pseudotime trajectory analysis confirmed that TAMs transition continuously between M1 and M2 phenotypes (Fig. 3a). The transcription factors IRF2, IRF7, IRF9, STAT2, and IRF8 seem important in determining TAMs differentiation [120] and could be utilized as epigenetic targets to induce M1 polarization of TAMs, resulting in proinflammatory and antitumor microenvironments.

T-cell phenotypes were determined using environmental stimuli and antigen T-cell receptor (TCR) stimulation. The overlap of TCR repertoires between cells in different states, known as TCR sharing, can also be utilized to study the evolution of T cells. Combining scRNA-seq and TCR tracking in colorectal cancer identified 20 T-cell subsets with distinct functions [109]. An exhaustion signature of 28 genes, including TIGIT, TNFRSF9/4-1BB, and CD27, was identified in exhausted T cells in melanoma tumours and was also found to be upregulated in high-exhaustion cells in most tumours [121]. Another study on T cells further identified other exhaustion markers in CD8^+^ T cells, such as LAYN, pleckstrin homology like domain family A member 1 (PHLDA1), and Synaptosome associated protein 47 (SNAP47) [108]. Pseudotime trajectory analysis indicated that T cells are in continuous activation and terminal differentiation (exhaustion) states in the TIME (Fig. 3b) [104]. Additional studies have been performed to study the evolution of exhausted T cells and potential targets to reverse T-cell exhaustion. scRNA-seq combined with TCR analysis demonstrated that dysfunctional exhausted T cells and cytotoxic T cells might be developmentally related in the TIME [122].

Consequently, studies have focused on the transition process of CD8^+^ T cells from effector to exhausted T cells [108]. Two CD8^+^ T-cell clusters were identified by scRNA-seq as pre-exhausted T cells in non-small cell lung cancer (NSCLC). The pre-exhausted to exhausted T cell ratio is associated with a better prognosis in lung adenocarcinoma. Thus, interrupting pre-exhausted T cells before exhaustion may be essential for cancer immunotherapy [123].

Due to the close interactions between immune cells and malignant cells, the evolution of malignant cells also plays a crucial role in immune cell evolution. Pseudotime trajectory analysis has indicated that the trajectory branch of metastatic lung adenocarcinoma is distinct from normal differentiation towards ciliated cells and alveolar-type cells [124]. Affected by the evolution of malignant cells, normal resident myeloid cell populations are replaced with monocyte-derived macrophages and novel dendritic cells (CD163^+^ CD14^+^ DCs). T cells have also been found to undergo exhaustion, constructing an immunosuppressive tumour microenvironment [124]. Similarly, another study demonstrated that anaplastic thyroid cancer cells are derived from a subcluster of papillary thyroid cancer cells, where a distinct TIME was constructed, leading to significantly worse prognoses [125].

### Communications between various cells in the TIME

**Fig. 4:**
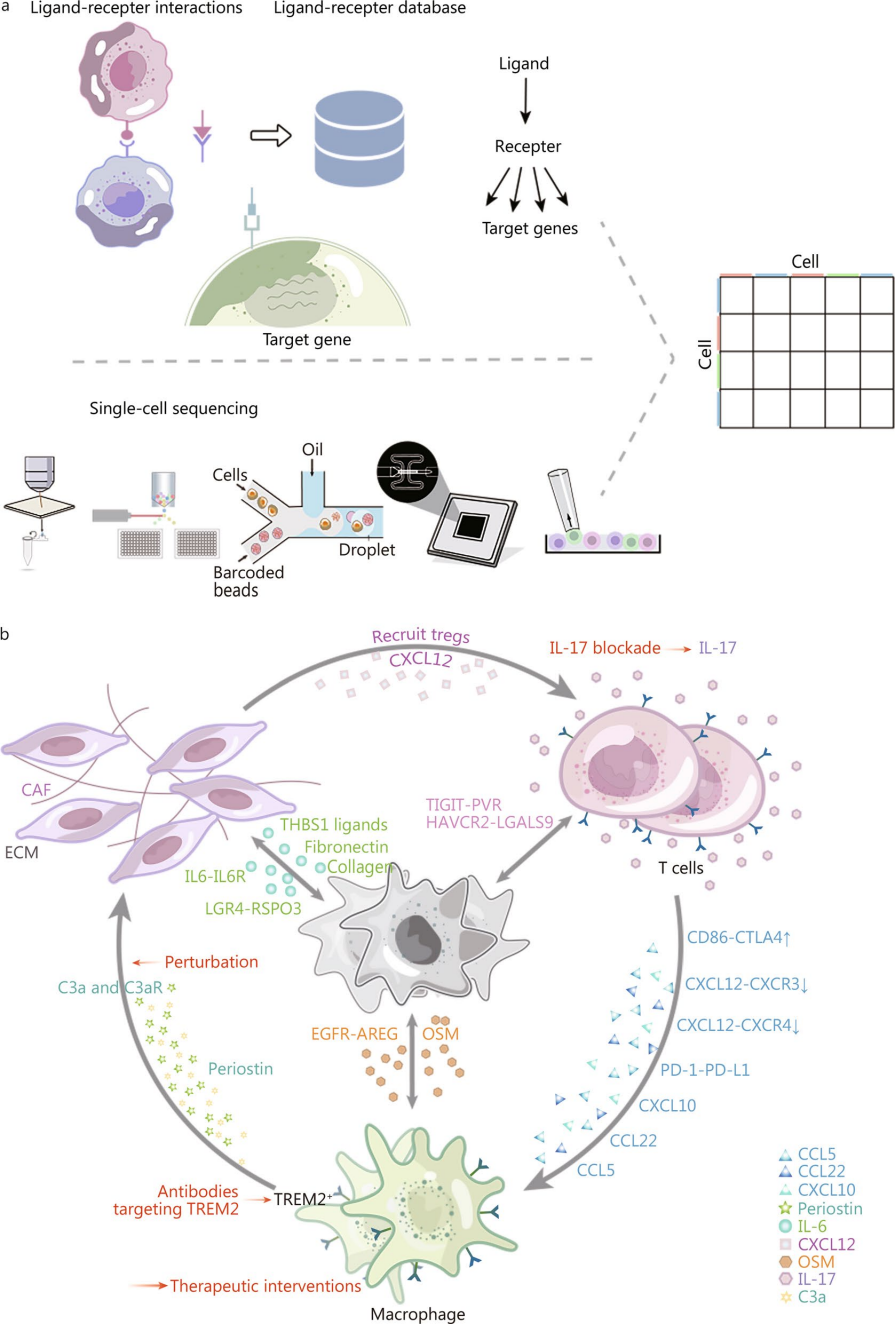
Cell communication between various cells in the tumour immune microenvironment. **a** Principles of analytical tools for investigating ligand-receptor interactions based on scRNA-seq data. These tools take advantage of databases of known ligand-receptor pair interactions. Some tools also consider the alterations in downstream pathways in receiver cells. **b** Malignant cells closely interact with immune cells. Tumour-associated macrophages (TAMs) were found to interact with malignant cells via the epidermal growth factor receptor (EGFR)-amphiregulin (AREG) ligand-receptor pair. oncostatin M (OSM) derived from TAMs also interacts with its receptor on malignant cells. T cells and malignant cells interact through T cell immunoreceptor with Ig and ITIM domains (TIGIT)-PVR and hepatitis A virus cellular receptor 2 (HAVCR2)-galectin 9 (LGALS9). CAFs and malignant cells interact through interlukin (IL)-6-IL6R, integrin receptor interactions with collagen and fibronectin, thrombospondin 1 (THBS1) ligands, and leucine rich repeat containing G protein-coupled receptor 4 (LGR4)-R-spondin 3 (RSPO3). Interactions between immune cells in the TIME have also been demonstrated. Cancer-associated fibroblasts (CAFs) recruit Tregs by secreting C-X-C motif chemokine ligand 12 (CXCL12) and are also correlated with M2 macrophages via periostin. TAMs show decreased CXCL12- C-X-C motif chemokine receptor 3 (CXCR3) and CXCL12- C-X-C motif chemokine receptor 4 (CXCR4) interactions and enhanced CD86-CTLA-4 and programmed death-ligand 1 (PD-L1)-programmed death-1 (PD-1) interactions with T cells. TAMs also secrete CXCL10, CCL22, and CCL5 to recruit T cells. TAMs and CAFs interact through C3a and C3aR. Therapeutic interventions, such as perturbation of complement C3a (C3a) and complement C3a receptor (C3aR), antibodies targeting triggering receptor expressed on myeloid cells 2 (TREM2), and IL-17 blockade, have displayed promising outcomes. TAMs tumour-associated macrophages, EGFR epidermal growth factor receptor, AREG amphiregulin, OSM oncostatin M, TIGIT T cell immunoreceptor with Ig and ITIM domains, HAVCR2 hepatitis A virus cellular receptor 2, LGALS9 galectin 9, IL interlukin, THBS1 thrombospondin 1, LGR4 leucine rich repeat containing G protein-coupled receptor 4, RSPO3 R-spondin 3, CAFs cancer-associated fibroblasts, CXCL C-X-C motif chemokine ligand, CXCR C-X-C motif chemokine receptor, PD-L1 programmed death-ligand 1, PD-1 programmed death-1, C3a complement C3a, C3aR complement C3a receptor, ECM extracellular matrix, TREM2 triggering receptor expressed on myeloid cells 2, CCL C–C motif chemokine ligand

Cell communication in the TIME is related to tumour progression. Ligand-receptor interactions, a vital type of cell communication, are essential for constructing the TIME and identifying potential therapeutic targets [126]. scRNA-seq was conducted on a cellular basis, making it feasible to investigate undiscovered cellular interactions. Many analytical tools for investigating ligand-receptor interactions based on scRNA-seq data have been developed (Fig. 4a), including iTALK [127], CellTalker [128], and CellPhoneDB [129]. These tools take advantage of databases of known ligand-receptor pair interactions. Among them, CellTalker utilizes differentially expressed genes, while CellPhoneDB includes the subunit architectures of ligands and receptors. Other tools, such as NicheNet, also consider the alterations in downstream pathways in receiver cells [130]. Other reviews have presented a detailed comparison of different computational approaches [131, 132].

During tumour progression, malignant cells lead to the recruitment and dysfunction of immune cells, which reciprocally influence tumorigenesis and the evolution of malignant cells [133], forming a vicious cycle (Fig. 4b). TAMs were found to interact with malignant cells via the epidermal growth factor receptor (EGFR)-amphiregulin (AREG) ligand-receptor pair. Modulation of AREG in a basal-like breast cancer cell line led to recruitment of anti-inflammatory TAMs [134]. Meanwhile, an EGFR-associated feedback loop was discovered to promote pancreatic adenosquamous carcinoma progression based on single-cell transcriptomics [135]. Oncostatin M (OSM) derived from TAMs also interacts with its receptor on malignant cells to activate signal transducer and activator of transcription 3 (STAT3) [136]. Researchers discovered communications between CAFs and gastric cancer cells via integrin receptor interactions with collagen, fibronectin, thrombospondin 1 (THBS1) ligands, and leucine rich repeat containing G protein-coupled receptor 4 (LGR4)- R-spondin 3 (RSPO3), which regulate stemness [110]. In addition, scRNA-seq of pancreatic ductal adenocarcinoma revealed interactions between TIGIT and hepatitis A virus cellular receptor 2 (HAVCR2) in T and NK cells, as well as their corresponding ligands PVR and LGALS9 in malignant cells, resulting in immune cell dysfunction and pancreatic cancer progression [137]. Hence, exploring cellular interactions between immune cells and malignant cells based on single-cell data offers possible therapeutic targets to disrupt the vicious cycle of tumour progression.

In addition to malignant cells, scRNA-seq and subsequent analysis also predicted the interactions between immune cells in the TIME, which presented opposite functions (Fig. 4b). For instance, TAMs were found to have decreased CXCL12- C-X-C motif chemokine receptor (CXCR)3 and CXCL12-CXCR4 interactions and enhanced CD86-CTLA-4 interactions between cytotoxic T cells and Tregs in nasopharyngeal carcinoma, resulting in a TIME that aggravates cancer progression [138]. In addition, CAFs recruit Tregs by secreting CXCL12 and are correlated with M2 macrophages via periostin [139]. In murine melanoma, researchers found that myeloid populations displayed the function of T cells recruitment via cytokine-receptor signals, including CXCL10, CCL22, and CCL5, and suppressed T-cell function via the programmed death-ligand 1 (PD-L1)-PD-1 axis [140]. On the other hand, some interactions between immune cells can induce an antitumor TIME. NK cells that recruit cDC1 cells via the chemokine receptor X-C motif chemokine receptor 1 (XCR1) were identified and found to promote cancer immune control [140, 141]. Thus, perturbation of interactions between immune cells might reconstruct the TIME, possibly slowing tumour growth.

The spatial location of cells is vital for cell communication. The application of scRNA-seq in studying physical interactions is limited because of the destructive process of tissue dissociation. Computational tools involving Cellular Spatial Organization mapper (CSOmap) have been presented to recapitulate the spatial organization of cells in the TIME [142]. Sequencing of physically interacting cells (PIC-seq) also helped in better depicting cell interactions. With the help of PIC-seq, Tregs have been discovered as a major T-cell type that interacts with DCs, suggesting that Treg-DCs interactions are important for sustaining an immune tolerance environment [143]. With the development of spatial transcriptomics techniques [144], the data of transcriptomics and spatial locations of cells are combined. Integrating single-cell and spatial transcriptomics helps us explore cell communications at a new level [145, 146]. However, spatial transcriptomics cannot reach the single-cell level. In situ transcriptomics at single-cell resolution might become possible in the future. Although commercially available methods, including Visium, cannot achieve the single-cell level, recent technologies enable transcriptomics research at single-cell or even subcellular resolution [147, 148]. Additionally, cell interactions occur at the protein level. scRNA-seq-based interaction predictions may not be mirrored accurately because scRNA-seq cannot directly reflect protein levels. Fortunately, single-cell proteomics and multiomics techniques have advanced dramatically [149, 150], which helps researchers better characterize cell communications.

### Clinical application and potential targets in the TIME based on scRNA-seq

Quantification of the TIME has been adopted in clinical practice for decades to predict patient survival and response to treatments. The immunohistochemistry-based immune score, which quantifies in situ immune cell infiltration in tumours, was proposed before scRNA-seq. The immune score is a better prognostic factor than the TNM classification in colorectal cancer [151]. The immuno-score is also associated with responses to different treatments [152], which emphasizes the essential role of the TIME in clinical applications.

Compared with the traditional immuno-score, scRNA-seq provides an unprecedented resolution of infiltrated immune cells in the TIME. Novel immune cell clusters related to prognosis have been identified. For instance, a distinct phenotype of low cytotoxic innate-like CD8^+^ T cells has been identified in early relapse hepatocellular carcinoma. These T cells overexpress KLRB1 while downregulating costimulatory and exhaustion-related molecules, including tumor necrosis factor receptor superfamily, member 9 (TNFRFS9), CD28, inducible T cell co-stimulator (ICOS), TIGIT, CTLA-4, and HAVCR2. The infiltration of this cluster of T cells is correlated with a poor prognosis in liver cancer [153]. In addition, scRNA-seq-based cellular interactions were also counted in the prediction model. Machine learning models have been constructed based on intercellular communication-associated genes (ICAGs) to predict the recurrence of lung adenocarcinoma. Combining eight ICAGs and patients' clinical information achieved an area under the receiver operator characteristic (ROC) curve of 0.841 [154]. In addition to prognosis prediction, unique cellular interactions in the TIME are also related to responses to immunotherapy. scRNA-seq analysis found distinct cell–cell communication networks between responders and non-responders to anti-PD-1 therapy, potentially predicting patient response to anti-PD-1 therapy [155]. As a result, patient prognosis and responses to immunotherapy were more precisely predicted with the help of scRNA-seq.

Taking advantage of scRNA-seq is inspiring in precision medicine, such as assisting targeted therapy to overcome drug resistance. For instance, physicians applied scRNA-seq of patient-derived xenografts (PDXs) before and after treatment in non-CR muscle-invasive bladder cancer patients treated with tipifarnib. Upregulation of PD-L1 was found in post-treatment PDXs and reduced the antitumor effects of immune cells. Accordingly, additional treatment with a PD-L1 inhibitor (atezolizumab) was chosen. Subsequently, patients achieved favourable responses [156]. In addition, novel immune subtypes were identified via scRNA-seq in monotherapy-resistant tumours. Blocking TAMs with anti-colony stimulating factor 1 receptor (CSF1R) failed to decrease tumour progression in cholangiocarcinoma. scRNA-seq identified compensatory enrichment of granulocytic myeloid-derived suppressor cells expressing APOE, which mediated T-cell inhibition. Dual inhibition of TAMs and granulocytic-Myeloid-derived suppressor cells (G-MDSCs) combined with anti-CSF1R and anti-lymphocyte antigen 6 complex, locus G (Ly6G) therapy augmented immune checkpoint blockade efficacy in a murine model, which is promising for clinical practice [157].

In addition to treating drug-resistant tumours, the application of scRNA-seq in the TIME has also highlighted potential novel targets that require further investigation. T cells are the most essential immune cells for removing malignant cells in the TIME. However, exhausted CD8^+^ T cells contribute to an unfavourable prognosis in different tumours. In addition to well-known immunosuppressive checkpoints, scRNA-seq identified exhausted CD8^+^ T cells highly expressing premelanosome protein (PMEL), tyrosinase related protein 1 (TYRP1), and endothelin receptor type B (EDNRB), which could serve as novel potential targets [158]. Myeloid cells are essential for recruiting immune cells in the TIME [159]. TREM2/APOE/complement component 1, q subcomponent (C1Q)-positive macrophage infiltration was identified by scRNA-seq as a prognostic biomarker for clear cell renal carcinoma recurrence [160]. Another study confirmed that antibodies targeting TREM2 in mice were associated with scant MRC1^+^ and CX3CR1^+^ macrophages and an expansion of myeloid clusters expressing immunostimulatory molecules, which promoted T-cell responses and led to a better prognosis [161]. Cellular interactions can also be used as therapeutic targets. scRNA-seq of intrahepatic cholangiocarcinoma revealed crosstalk between vCAFs and intrahepatic cholangiocarcinoma (ICC) cells. IL-6 secreted by vCAFs induces epigenetic alterations in ICC cells, which enhance malignancy [162]. Hence, the interruption of IL-6 signaling in ICC has become quite intriguing. Potential targets for cancer therapy as indicated by scRNA-seq are summarized in Table 2.

**Table 2:** Potential targets in the TIME indicated by scRNA-seq

Potential targets	Mechanisms	Potential targets	Therapeutic intervention
Malignant cells	Inactivation of antigen presentation	ADAR1 [163]	–
	Depletion and dysfunction of CD8^+^ T cells	MUC1-C [164]	–
	Macrophage exhaustion	PDIA5 [165]	–
	Inhibition of ICOSL^+^ B-cells	CD55 [166]	–
Exhausted CD8^+^ T cells	Dysfunction of T cells	PMEL, TYRP1, and EDNRB [158]	–
CD8^+^ T cells	Transcriptional regulators determining T cell fates	FOXO1, KDM5B, MAF, IKZF2, SOX4, and BCL3 [110]	–
NK cells	Inhibitory and costimulatory molecules	TNFRSF18 (GITR), CD96, and KIR2DL4 [110]	–
	HIF-1α, reducing antitumor effects	HIF-1α inhibitor [167]	–
Immature myeloid cells	Immune suppression	–	Tyrosine kinase inhibitor cabozantinib [168]
TREM2^+^ TAMs	Associated with T-cell response	–	Antibodies targeting TREM2 [160, 161]
PD-L1^+^ TAMs and APOE^+^ G-MDSCs	T-cell inhibition induced by TAMs and G-MDSCs	–	anti-CSF1R and anti-Ly6G antibodies [157]
vCAFs/ICC	Enhancement of malignancy	IL-6 [162]	–
CAFs/malignant cells	Immune evasion	PVR-TIGIT, LGALS9-HAVCR2, and TGF-β-TGF-βR axis [169]	–
PLVAP/VEGFR2 ECs/FOLR2 TAMs	Onco-foetal reprogramming of the tumour ecosystem	VEGF/NOTCH [170]	–
Malignant cells/IL-17-producing T cells	Activation of angiogenesis and suppression of CD8^+^ T cells	–	IL-17 blockade [171]
CXCL13^+^ T cells/B cells	Associated with prognosis	CXCL13/CXCR5 [172]	–

“–” no evidence applied by studies, *TIME* tumour immune microenvironment, *NK* natural killer, *TREM2* triggering receptor expressed on myeloid cells 2, *TAMs* tumour-associated macrophages, *PD-L1* programmed cell death-Ligand 1, *APOE* apolipoprotein E, *G-MDSCs* granulocytic-myeloid-derived suppressor cells, *CAFs* cancer-associated fibroblasts, *vCAFs* vascular CAFs, *ICC* intrahepatic cholangiocarcinoma, *PLVAP* plasmalemma vesicle associated protein, *VEGFR2* vascular endothelial growth factor receptor 2, *ECs* endothelial cells, *FOLR2* folate receptor beta, *ICOSL* inducible T-cell co-stimulator ligand, *HIF-1α* hypoxia induced factor-1α, *ADAR1* adenosine deaminase RNA specific 1, *MUC1-C* mucin 1 C-terminal subunit, *PDIA5* protein disulfide isomerase family A member 5, *PMEL* premelanosome protein, *TYRP1* tyrosinase related protein 1, *EDNRB* endothelin receptor type B, *FOXO1* forkhead box O1, *KDM5B* lysine demethylase 5B, *MAF* macrophage activating factor, *IKZF2* IKAROS family zinc finger 2, *SOX4* SRY-box transcription factor 4, *BCL3* β-cell CLL/lymphoma 3, *TNFRSF18* TNF receptor superfamily member 18, *TIGIT* T cell immunoreceptor with Ig and ITIM domains, *HAVCR2* hepatitis A virus cellular receptor 2, *VEGF* vascular endothelial growth factor, *TGF-β* transforming growth factor beta, *TGF-βR* transforming growth factor beta receptor, *IL-6* interleukin 6

### Application of scRNA-seq in the future

Even though the application of scRNA-seq has made great progressions in TIME studies, the promotion and popularization of single-cell sequencing technology are limited by high sample quality requirements, limited throughput, inevitable technical errors and high costs [173]. It is hoped that with the development of scRNA-seq technology, the threshold will become lower, accelerating the widespread application of scRNA-seq. Currently, single-cell analysis has been extended beyond transcriptomics to genomics, proteomics, and epigenetics [149, 174, 175]. The actual spatial structure of cells in tumour tissues can be reconstructed [176]. Moreover, frozen specimens and paraffin-embedded tissues can be analysed in addition to fresh tissues [177, 178]. These technique improvements shed light on construction of large single-cell datasets with high resolution.

The application of single-cell analysis to precision medicine is promising. Several studies have revealed the substantial value of using scRNA-seq in clinical practice. scRNA-seq of the skin and blood of a patient with drug-induced hypersensitivity syndrome/drug reaction with eosinophilia and systemic symptoms (DiHS/DRESS) identified central memory CD4^+^ T cells enriched in human herpesvirus 6b DNA and the Janus kinase-signal transducer and activator of transcription (JAK-STAT) signaling pathway as potential targets. Subsequent treatment with tofacitinib and antiviral agents was successful in this individual patient [179]. scRNA-seq also provides potential targets for cancer therapy. Increased intratumoral heterogeneity was discovered in therapy-resistant small-cell lung cancer, emphasizing the importance of combination therapies for treatment-naïve tumours [180].

## Conclusions

The unprecedented power of scRNA-seq has started a new era in TIME research. A comprehensive cellular atlas of the TIME has been drawn, providing a novel perspective for clinical application in various tumours. In addition, cellular compositions and communications in the TIME provide potential targets for cancer therapies and contribute to the development of precision medicine. We believe that advances in technology and wide application of single-cell analysis can lead to the discovery of novel perspectives on cancer therapy and that scRNA-seq can subsequently be implemented more frequently in clinical practice.

## Data Availability

Not applicable.

## Abbreviations

ADAR1: Adenosine deaminase RNA specific 1
apCAFs: Antigen-presenting CAFs
APOE: Apolipoprotein E
AREG: Amphiregulin
BCL3: β-Cell CLL/lymphoma 3
BHLHE40: Basic helix-loop-helix family member E40
C1Q: Complement component 1, q subcomponent
C3a: Complement C3a
C3aR: Complement C3a receptor
CAFs: Cancer-associated fibroblasts
cCAFs: Cycling CAFs
CCL: C–C motif chemokine ligand
cDC: Conventional DC
cDNA: Complementary DNA
CSF1R: Colony stimulating factor 1 receptor
CSG: Cell surface glycoprotein
CSOmap: Cellular Spatial Organization mapper
CTLA-4: Cytotoxic T lymphocyte-associated antigen-4
CXCL: C-X-C motif chemokine ligand
CXCR: C-X-C motif chemokine receptor
dCAFs: Developmental CAFs
DCs: Dendritic cells
DiHS/DRESS: Drug-induced hypersensitivity syndrome/drug reaction with eosinophilia and systemic symptoms
ECs: Endothelial cells
EDNRB: Endothelin receptor type B
EGFR: Epidermal growth factor receptor
EMT: Epithelial-mesenchymal transition
FACS: Fluorescence-activated cell sorting
FCN1: Ficolin 1
FOLR2: Folate receptor beta
FOXO1: Forkhead box O1
G-MDSCs: Granulocytic-Myeloid-derived suppressor cells
GZMK: Granzyme K
HAVCR2: Hepatitis A virus cellular receptor 2
HIF-1α: Hypoxia induced factor-1α
iCAFs: Inflammatory CAFs
ICAGs: Intercellular communication-associated genes
ICC: Intrahepatic cholangiocarcinoma
ICOS: Inducible T cell co-stimulator
ICOSL: Inducible T-cell co-stimulator ligand
IDO1: Indoleamine 2,3-dioxygenase 1
IFN-γ: Interferon-γ
IKZF2: IKAROS family zinc finger 2
IL: Interleukin
ISG: Interferon-stimulated genes
IVT: In vitro transcription
JAK: Janus kinase
KDM5B: Lysine demethylase 5B
KIR: Killer cell immunoglobulin like receptor
LAG3: Lymphocyte activating 3
LAMP3: Lysosomal associated membrane protein 3
LAYN: Layilin
LCM: Laser capture microdissection
LGALS9: Galectin 9
LGR4: Leucine rich repeat containing G protein-coupled receptor 4
Ly6G: Lymphocyte antigen 6 complex, locus G
MAF: Macrophage activating factor
MARCO: Macrophage receptor with collagenous structure
mCAFs: Matrix CAFs
MHC: Major histocompatibility complex
MS4A6A: Membrane spanning 4-domains A6A
MUC1: Mucin1
MUC1-C: Mucin 1 C-terminal subunit
myCAFs: Myofibroblastic CAFs
NK: Natural killer
NKT: Natural killer T
NSCLC: Non-small cell lung cancer
OSM: Oncostatin M
PCR: Polymerase chain reaction
PD-1: Programmed death 1
PDIA5: Protein disulfide isomerase family A member 5
PD-L1: Programmed death-ligand 1
PDX: Patient-derived xenografts
PHLDA1: Pleckstrin homology like domain family A member 1
PIC-seq: Sequencing of physically interacting cells
PLAUR: Plasminogen activator, urokinase receptor
PLVAP: Plasmalemma vesicle associated protein
PMEL: Premelanosome protein
ROC: Receiver operator characteristic
RSPO3: R-spondin 3
scRNA-seq: Single-cell RNA sequencing
SNAP47: Synaptosome associated protein 47
SOX4: SRY-box transcription factor 4
SPP1: Secreted phosphoprotein 1
STAT: Signal transducer and activator of transcription
TAMs: Tumour-associated macrophages
TCF7: Transcription factor 7
TCR: T-cell receptor
TGF-β: Transforming growth factor beta
TGF-βR: Transforming growth factor beta receptor
Th1: T helper 1
Th17: T helper 17
Th2: T helper 2
THBS1: Thrombospondin 1
TIGIT: T cell immunoreceptor with Ig and ITIM domains
TIME: Tumour immune microenvironment
TLSs: Tertiary lymphoid structures
TNFRSF18: TNF receptor superfamily member 18
TNFRSF9: TNF receptor superfamily member 9
TNF-α: Tumor necrosis factor-α
Tregs: Regulatory T cells
TREM2: Triggering receptor expressed on myeloid cells 2
TYRP1: Tyrosinase related protein 1
UMI: Unique molecular identifier
vCAFs: Vascular CAFs
VEGF: Vascular endothelial growth factor
VEGFR2: Vascular endothelial growth factor receptor 2
XCR1: X-C motif chemokine receptor 1
